# All Good Things Must End: Termination of Receptor Tyrosine Kinase Signal

**DOI:** 10.3390/ijms22126342

**Published:** 2021-06-14

**Authors:** Azzurra Margiotta

**Affiliations:** 1 Department of Biology, Faculty of Medicine, Masaryk University, 62500 Brno, Czech Republic; azzurra.margiotta@med.muni.cz; 2 International Clinical Research Center, St. Anne’s University Hospital, 65691 Brno, Czech Republic

**Keywords:** RTKs, FGFRs, termination of signaling, degradation, ubiquitination, PTPs, kinases

## Abstract

Receptor tyrosine kinases (RTKs) are membrane receptors that regulate many fundamental cellular processes. A tight regulation of RTK signaling is fundamental for development and survival, and an altered signaling by RTKs can cause cancer. RTKs are localized at the plasma membrane (PM) and the major regulatory mechanism of signaling of RTKs is their endocytosis and degradation. In fact, RTKs at the cell surface bind ligands with their extracellular domain, become active, and are rapidly internalized where the temporal extent of signaling, attenuation, and downregulation are modulated. However, other mechanisms of signal attenuation and termination are known. Indeed, inhibition of RTKs’ activity may occur through the modulation of the phosphorylation state of RTKs and the interaction with specific proteins, whereas antagonist ligands can inhibit the biological responses mediated by the receptor. Another mechanism concerns the expression of endogenous inactive receptor variants that are deficient in RTK activity and take part to inactive heterodimers or hetero-oligomers. The downregulation of RTK signals is fundamental for several cellular functions and the homeostasis of the cell. Here, we will review the mechanisms of signal attenuation and termination of RTKs, focusing on FGFRs.

## 1. Introduction

RTKs are a class of membrane spanning cell-surface receptors that are characterized by an intrinsic protein tyrosine kinase activity. In fact, they catalyze the transfer of the γ phosphate of ATP to hydroxyl groups of tyrosines to target proteins. Therefore, RTKs are important mediators of intracellular signaling and regulate the cell cycle, cell proliferation, cell migration, cell differentiation, apoptosis, fertilization, cell metabolism, and survival [1]. In humans, 58 RTKs have been discovered and have been classified into 20 subfamilies. The structure of RTK proteins is highly conserved as they share an N-terminal ligand-binding extracellular domain (ECD), composed generally of several hundred amino acids and normally glycosylated, a single-pass transmembrane helix (TMD), an intracellular juxtamembrane domain (JMD), a tyrosine kinase domain (KD) that is normally 275 amino acids long, and a C-terminal tail domain [2]. One of the important RTKs are fibroblast growth factor receptors (FGFRs), which are coded by four genes (*FGFR1*, *FGFR2*, *FGFR3*, and *FGFR4*), and 18 fibroblast growth factors (FGFs) related to the receptors are known nowadays. FGFRs contain in the extracellular domain three immunoglobulin (Ig)-like domains (D1-D3). Moreover, they are characterized by the presence of a highly conserved motif rich in aspartate acids, called the acid box. Interestingly, the extracellular domain of FGFRs has been proved to have an auto-inhibitory function [1,3,4].

RTKs are located at the plasma membrane (PM) where they are in monomeric form. Their function is based on their ability to bind extracellular ligands and to form dimers or oligomers that show active intracellular kinase domains and determine receptor phosphorylation. In fact, the contact between the KDs of dimers stimulates catalytic activity, the auto-phosphorylation, and the cross-phosphorylation of receptor molecules and substrates [2]. Therefore, this leads to the initiation of downstream signaling cascades that are fundamental for several cell biological functions [5]. In fact, several molecules such as SHC, SRC, phospholipase Cγ, STAT1, Gab1, and FRS2α are recruited after phosphorylation of the docking sites, and the Ras-Raf-Erk 1/2 and the PI3K-Akt pathways are activated [6]. Intriguingly, individual RTKs can bind several ligands that can induce differently the dimerization of the receptor [1]. For instance, FGFRs are able to bind several fibroblast growth factors (FGFs). In particular, 22 FGF ligands have been discovered in humans where FGF1 and FGF2 belong to the FGF1 subfamily, FGF4, FGF5, and FGF6 are members of the FGF4 subfamily, and the FGF7 subfamily is composed of FGF3, FGF7, FGF10, and FGF22 while the FGF8 subfamily contains FGF8, FGF17, and FGF18. Furthermore, the FGF9 subfamily includes FGF9, FGF16, and FGF20, whereas FGF19, FGF21 and FGF23 belong to the FGF19 subfamily. However, four members of this family, FGF11, FGF12, FGF13, and FGF14 do not bind to FGFR and are considered FGF homologous factors. Interestingly, it has been proved that the effects due to the ligands FGF1 or FGF2 are very different and are based on the structural changes in the formation of the FGFR dimers that affect phosphorylation of the receptor. FGF signaling is important in several processes such as embryonic development and adult homeostasis, differentiation, and metabolism [3,7,8]. Therefore, regulation of FGFR signaling is fundamental for avoiding diseases such as cancer and developmental disorders.

**Figure 1:**
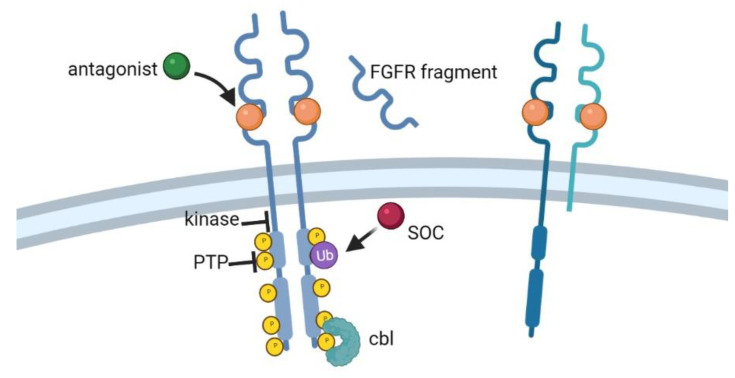
Mechanisms of attenuation and termination of RTK signaling. FGFR dimers and ligand binding are shown. On the left, several mechanisms of downregulation or termination of RTK signaling are shown, such as binding of antagonist molecules, kinase or PTP activity, SOC and CBL regulation of ubiquitination, and degradation of the receptor after internalization. On the right, RTK dimer is composed by the association of one full-length monomer and an RTK truncated form that lacks the KD, generating a dominant negative mutant.

The mechanisms that allow modulation of RTK activity and signaling are based on signal attenuation and termination [1]. RTK downregulation can occur through endocytosis and degradation of the receptor, expression of antagonistic ligands or of dominant negative deletion mutants that will generate inactive heterodimers or hetero-oligomers, and inhibition of RTK activity through the action of specific kinases, tyrosine phosphatases, or of negative regulators (Figure 1). Moreover, the trafficking of RTKs to subcellular compartments and their location are coupled to signal transduction [1,9]. Here, we will discuss the main mechanisms of downregulation of RTKs, with a special focus on FGFRs.

## 2. Ubiquitination-Dependent Lysosomal Degradation of RTKs

Receptor tyrosine kinase signaling can be terminated by internalization of the receptor after ligand binding at the plasma membrane. This occurs after autophosphorylation of RTKs and phosphorylation of secondary signaling molecules that bind motifs containing phosphotyrosine residues, including phosphotyrosine binding (PTB) and Src-homology 2 (SH2) domains [10]. Firstly, endocytosis leads to downregulation of signaling by affecting the localization of the receptors, as it will not be at the PM anymore [11]. The internalization of RTKs may occur through clathrin-mediated endocytosis or clathrin-independent endocytosis. Once inside the cell, the signaling can continue from the endosomal compartments or other compartments, the receptor can be destined for lysosomes for degradation with termination of the signaling, or it can be recycled to the PM to potentiate further signaling. Interestingly, enrichment of RTKs within specific plasma membrane microdomains has been linked to the internalization of the receptor and regulates RTK signaling [10]. Concerning FGFR trafficking, signal transduction, and regulation of RTK signaling, Auciello and colleagues showed how these receptors, once activated, enter the cells through pre-formed clathrin-coated pits (CCPs); subsequently, they are localized on peripheral early endosomes (EEs) and directed to recycling endosomes or to degradative compartments (through multivesicular bodies (MVBs)/late endosomes (LEs), and subsequent fusion of these vesicles with lysosomes) regulated by the action of Eps8 and Src [12].

Once on intracellular vesicles, RTK downregulation is controlled by both ubiquitination and deubiquitination. Ubiquitination in the cytoplasmic domain of RTKs occurs at an early stage and is used as a sorting signal for their sorting and delivery to MVBs [13]. Ubiquitin is added to the target protein through sequential reactions catalyzed by three classes of enzymes (E1, E2, and E3) that determine the addition of the molecule of ubiquitin (Ub) to the ε-amino group of a lysine residue. Subsequent reactions can attach new molecules of Ub in order to make polyubiquitin chains [14]. The mechanism of action of the E3 ubiquitin ligase c-CBL is based on the phosphorylation of specific tyrosines of the RTK, the binding at this site of c-CBL, and its phosphorylation. Therefore, its ubiquitin ligase ability is activated, a ubiquitin-loaded E2 enzyme binds to the RING-finger domain of c-CBL, and the RTK is covalently tagged with Ub [15,16,17].

Mono-ubiquitination and multi-ubiquitination (mono-ubiquitination at several sites) are the signals for lysosomal degradation of membrane proteins, and several molecules take part in the regulation of RTK degradation, such as the ubiquitin ligase c-CBL or the endocytic regulator proteins Epsin, Eps15, Rabex5, Hrs, STAM, GGA3, and Tsg101 [18]. For instance, Eps15 is a ubiquitin-binding protein at CCPs which works in the receptor recruitment step together with Epsin and Hip1 [18,19]. The endocytic vesicle loses the clathrin coat and fuses with an EE. During this process Rab5, Rabaptin-5, Rabex5, and EEA1 are involved where Rab5 catalyzes the reaction, helped by its effector Rabaptin-5 and the Rab5 GDP/GTP exchange factor Rabex5. EEA1 functions as a tethering factor. EEs can fuse with other EEs or mature to MVBs and lysosomes [18].

Interestingly, ubiquitinated proteins at EEs are recognized by a complex of two ubiquitin-binding proteins, hepatocyte growth factor-regulated substrate (Hrs) and signal-transducing adaptor molecule (STAM), which belong to the class E Vps (vacuolar protein sorting) proteins and form ESCRT-0. The proteins coded by the VPS genes are fundamental for the normal membrane trafficking to lysosomes. RTKs are then transferred to the endosomal sorting complex required for transport (ESCRT)-I and ESCRT-II and then transported to MVBs with the help of ESCRT-III [13]. It has been discovered that mutations in the genes for class E Vps determine alterations of the RTK signaling [20,21,22,23,24]. Finally, RTKs are transported to lysosomes and degraded due to the abundant acid hydrolases [25].

Deubiquitination enzymes (DUBs) are proteases which specifically hydrolyze the isopeptide bond between Ub and a determined protein or between Ub molecules in case of Ub chains. This class of proteins includes Ub-specific protease Y (UBPY) and associated molecule to the SH3 domain of STAM (AMSH) in mammals and Doa4 in yeast that associates with class E Vps proteins on endosomes. UBPY and AMSH interact with the Hrs-STAM complex and counteract ubiquitination of RTKs. The specific roles of DUBs are to process the newly synthesized Ub precursors in order to generate monomeric Ub, retrieving Ub from proteasome- and lysosome-targeted poly- and mono-ubiquitinated proteins before degradation, disassembling poly-Ub chains into Ub monomers, and counteracting the ubiquitination-dependent regulatory process [26,27].

### Degradation of FGFRs

**Figure 2:**
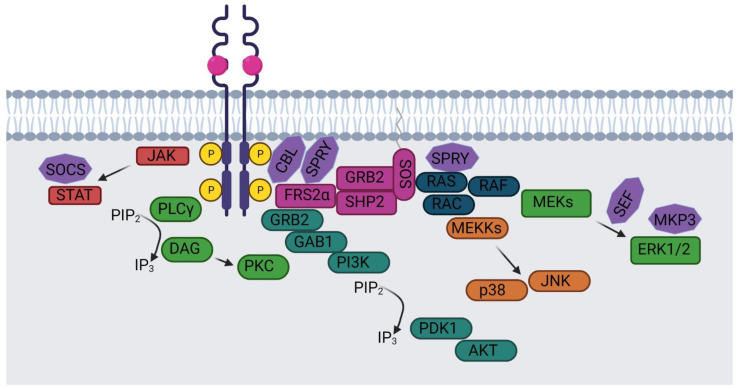
FGFR signaling cascade and inhibitory molecules. FGF receptor modulates several pathways in the cell through the action of many molecules. Inhibitors of FGFR signaling cascade can act on different levels and on several steps of this process. Molecules indicated by a violet star represent the proteins able to negatively regulate the activity of the FGFR.

FGFRs compose a family of four single-pass transmembrane receptors, FGFR1, FGFR2, FGFR3, and FGFR4. A fifth FGFR-like protein, FGFR5/FGFRL1, lacks the KD necessary for the intracellular signaling; however it can dimerize with other FGFRs [28,29,30]. After dimerization, phosphorylation, and internalization, FGFR4 is normally directed back to the PM through recycling endosomes, whereas FGFR1-3 are generally sorted to lysosomes for degradation. Moreover, FGFR1 is the most ubiquitinated FGFR and the one fastest degraded, indicating therefore a correlation between ubiquitination level and degradation [31]. The termination and negative regulation of the FGFR signaling cascade through internalization and degradation of FGFRs is mediated by the ubiquitin ligase CBL [28] (Figure 2). CBL is an E3 ubiquitin ligase that triggers the down-regulation, a major deactivation pathway, of both RTKs and nonreceptor tyrosine kinases [32,33,34].

Once the FGFR is activated, a ternary complex formed of FRS2α, GRB2, and CBL is formed. In fact, the binding of the ligand FGF to FGFR determines the phosphorylation of FRS2, its binding to GRB2, and the recruitment of CBL, which is responsible for ubiquinating the FGFR and FRS2 [35]. Interestingly, a mutated form of CBL, CBL G306E, did not interact with FGFR2 and inactivated the PTB domain, resulting in a loss of function and downregulation of the signal. This affected osteogenic differentiation in human bone marrow-derived mesenchymal stromal cells. In fact, the activation of FGFR2 and the presence of the mutant of CBL determined less FGFR2 ubiquitination and degradation upon activation by ligand, therefore inducing higher ERK1/2 and PI3K phosphorylation, whereas the post-translational modification of Akt was not affected [36,37,38,39].

Another mechanism of modulation of FGFR activity that involves FRS2α, GRB2, and CBL regards Sprouty (Spry) proteins. Spry proteins inhibit signaling of the RTK when, after stimulation by growth factors, they reach the PM, become phosphorylated on a conserved tyrosine, and bind to GRB2, therefore inhibiting the recruitment of GRB2-SOS complex either to FRS2 or to Shp2. Intriguingly, the phosphorylated Spry2 inhibited GRB2 from binding FRS2 or Shp2 and blocked ERK signaling. When a dominant negative mutant of Spry was expressed, which was not phosphorylated, a prolonged activation of ERK signaling and relative induced outgrowth of neurites in PC12 cells was detected [40]. Interestingly, CBL interacts with Spry proteins, and it has been proved that Spry2 was tyrosine-phosphorylated upon stimulation by FGFR and subsequently bound to c-CBL with high affinity, interfering with its activity as it competed for the ring finger domain of c-CBL with the E2-ubiquitin ligase. While this seemed relevant for EGFR downregulation, the conjugation of Ub to FGFR1 was not influenced by Spry2. Nevertheless, the interaction between c-CBL and Spry2 was fundamental for the inhibition of FGFR1-stimulated ERK activity. This was due to the ability of c-CBL to act as a docking protein for other proteins rather than its ubiquitination activity. Therefore, the interaction between c-CBL and Spry2 was important for the formation of protein complexes that affected FGFR1 signaling. Interestingly, the mutated form of Spry2, Spry2Y55F, partially influenced the FGFR1 ubiquitination state [41]. Similarly, Spry affected the production of ectopic branches on the stalks of primary branches in the tracheal system in Drosophila [42]. Interestingly, both Drosophila Spry proteins (dSpry) and human Spry proteins (hSpry) have been detected to inhibit the Ras/mitogen-activated protein (MAP) kinase pathway. Indeed, Spry1, Spry2, and Spry4 took part in the signaling cascade which was started with FGFR activation, FRS phosphorylation and its association with the GRB2-SOS complex, and finally, the activation of the Ras/MAP kinase pathway [43]. 

## 3. Inhibition of RTK Activity

In relation to RTK signaling, variations in strength and duration of the signal affect cell functions. Signal termination is controlled by several molecules, and degradation after internalization of the receptor is not the only way to downregulate the activity of RTKs. The activity of molecules such as kinases and protein tyrosine phosphatases (PTPs) has a regulatory effect on RTKs [44]. Moreover, other proteins, such as suppressor of cytokine signaling (SOCS), adapter containing PH and SH2 domains (APS), Lnk, SH2-B, and receptor-associated late transducer (RALT) can act as negative regulators of RTK signaling [45].

### 3.1. Protein Phosphatases

Downregulation of signaling can occur also through the action of PTPs, which reverse RTK signals by dephosphorylating them. Seven autophosphorylation sites have been detected in FGFR1 (Y463, Y583, Y585, Y653, Y654, Y730, Y766) and FGFR2 (Y466, Y586, Y588, Y656, Y657, Y733, Y769). An eighth tyrosine (Y776 for FGFR1, Y779 for FGFR2, Y770 for FGFR3, Y764 for FGFR4) is conserved in all FGFRs. Besides this, five conserved tyrosine residues have been discovered in FGFR3 (Y577, Y647, Y648, Y724, Y760) and four in FGFR4 (Y642, Y643, Y719, Y754) [46].

A fine tuning of RTK signaling is exerted through a spatial and temporal restricted action of the PTP [44]. Moreover, the concentration of PTPs and RTKs determine the kinetic parameters of the receptor dephosphorylation and inactivation [47]. A total of 107 human genes coding for PTPs have been discovered [48,49]. PTPs can be classified as transmembrane proteins (receptor-like RPTPs) and non-transmembrane PTPs. Their action is regulated either by their recruitment to RTKs or by the modulation of specific PTP activity [44]. Nevertheless, PTPs can function both as oncogene, by enhancing RTK signaling, and as tumor suppressor, such as in the case of protein tyrosine phosphatase-1B (PTP1B), which is supposed to terminate EGFR signaling [50]. Generally, PTPs contain two intracellular, structurally different, phosphatase domains, the catalytically active membrane-proximal domain (D1) and the membrane-distal domain (D2), with no clear function [51]. Interestingly, dephosphorylation mediated by PTPs can be coupled together with internalization and degradation of the receptor [52]. Indeed, PTP1B is located at the endoplasmic reticulum (ER) while its targets, several RTKs including insulin receptor, epidermal growth factor (EGFR), platelet-derived growth factor receptors (PDGFR) and IGF1R, are on the PM when they become activated. Fluorescence resonance energy transfer (FRET) analysis demonstrated that RTKs, such as EGFR and PDGFR, are internalized and, once they have arrived at the ER, interact with PTP1B, from which they are dephosphorylated and inactivated [52]. Moreover, several PTPs could affect the same RTK as it has been demonstrated that individual PTP knockouts may not affect the receptor signaling phenotype [44,53,54]. In fact, on one side, PTP-1B, T cell -PTP, PTP-α, PTP-ε, and LAR regulate insulin receptor signaling [55,56,57,58,59,60,61] while, on the other side, knockout of LAR does not determine any effect on this RTK signaling [62].

Interestingly, the PTP CLR-1 (CLeaR) has been associated with a FGFR-related receptor discovered in *Caenorhabditis elegans*, which is named *EGL-15*. CLR-1 has a complex extracellular region and two intracellular phosphatase domains, and it downregulates the activity of egl-15 by dephosphorylating it [51]. Intriguingly, another PTP, named PTPRB, previously identified as positive regulator for vascular endothelial cadherin (VE-cadherin) and negative modulator for TEK, has been discovered to negatively affect FGFR2 activity in mice. PTPRB is a transmembrane protein tyrosine phosphatase that acts by regulating blood vessel remodeling and angiogenesis and, in particular, by modulating in a negative manner FGF2-dependent branching morphogenesis. It has been demonstrated that *Ptprb* is highly expressed in adult mammary stem cells and is responsible for branching morphogenesis in the mammary epithelium. Therefore, knockdown of PTPRB in mammary epithelial cells induces a higher level of FGFR2 activation and ERK1/2 phosphorylation [63].

Interestingly, another phosphatase that affects FGFR signaling is Mkp3. Its transcription is regulated by the activation of FGFRs, then it dephosphorylates MAPK and inhibits MAPK signaling by acting as negative feedback regulator of FGFR [64].

### 3.2. Kinases

RTK activity can be regulated through the modulation of the action of protein kinases in order to protect the cells from excessive intracellular signaling. Interestingly, in the case of FGFR1, FGF1 ligand binding determined the direct ERK1/2-mediated phosphorylation of a specific serine residue (Ser777) that is present in the C-terminal domain of the FGFR1. The phosphorylation of this site inhibited the phosphorylation of tyrosines in the KD through a negative feedback route. Therefore, FGFR1 activation induces a negative feedback loop via ERK1/2 signaling. Furthermore, inactivation of ERK1/2 abrogated the phosphorylation of FGFR1. Nevertheless, the abolition of the phosphorylation event or the mutation of the serine residue to alanine, which cannot be phosphorylated, determined enhanced FGFR1 activity after FGF1 ligand binding and increased cell proliferation, cell migration and axonal growth. On the contrary, mutation of Ser777 to a phosphomimetic residue reduced the FGFR1 signaling [65]. Interestingly, FGFR1 is phosphorylated at Ser777 also by the α isoform of p38 mitogen-activated protein kinase (MAPK) [66]. Moreover, MAPK signaling and threonine phosphorylation of FRS2α were also involved in another mechanism of attenuation of FGFR1 signaling. In fact, upon FGF stimulation and FGFR1 activation, the kinases ERK1 and ERK2 phosphorylated at least eight tyrosines of FRS2α, determining a reduced recruitment of GRB2 and contributing to FGFR1 downregulation of the signal [67].

### 3.3. Other Negative Regulators of RTK Signaling

Termination and fine-tuning of RTK signaling activity occur through different mechanisms and the involvement of other negative regulators such as SOCS, APS, Lnk, SH2-B, and RALT [45]. APS, Lnk and SH2-B are characterized by a common structural organization with an NH_2_-terminal region containing potential binding sites for SH3 domains, a PH domain, an SH2 module, and a COOH tail containing a conserved tyrosine phosphorylation site. They compose a family of structurally and functionally related adapters that share the role of negative regulators of RTKs and cytokine receptors [68]. For instance, APS expressed in human osteosarcoma cell lines becomes phosphorylated after stimulation of several growth factors, such as PDGF, insulin-like growth factor (IGF), and granulocyte-macrophage colony stimulating factor (GM-CSF). Phosphorylated APS decreased the activity of ERK2 induced by the activity of PDGFR through binding c-CBL [69].

Similarly, Src-like adapter protein (Slap) negatively affects the activity of PDGFR by competing with Src for binding and blocking Src-mediated mitogenic signaling [70]. Another example is RALT, which exhibits its negative regulatory activity on ErbB-2 [45]. RALT interacts with ErbB-2 and, through its action on GRB-2, regulates ErbB-2 mitogenic function, inhibiting its ERK1- and ERK2-dependent downstream signaling [71].

Among the proteins involved in tuning and terminating the receptor signaling, the family of SOCS proteins represents a set of proteins widely involved in the negative regulation of several RTKs [45]. SOCS family members bind elongin B, cullin, and Rbx1 to form an E3 ubiquitin ligase complex. This protein association is responsible for the addition of polyubiquitin chains and proteasome degradation. In particular, it is the ubiquitylation of tyrosine kinase receptor that is involved in the attenuation of the signal [72,73]. Interestingly, SOCS1 and SOCS3 interact with and modulate FGFR signaling. Indeed, it has been demonstrated that SOCS1 and SOCS3 co-immunoprecipitate, independently of the activation state of the RTK, and colocalize with the receptor. This protein association is responsible for the addition of polyubiquitin chains and proteasome/lysosome degradation. Moreover, FGFR3 and SOCS1 cluster together in the perinuclear cytosolic part of the cell. Furthermore, upon FGFR3 activation in cells overexpressing SOCS1, STAT1 phosphorylation was repressed while MAPK phosphorylation was increased and sustained. Moreover, SOCS1 overexpression led to FGFR3 degradation attenuation. Therefore, STAT1 phosphorylation was repressed while MAPK phosphorylation was high and sustained. This led to FGFR signaling attenuation [74]. 

Another inhibitor of FGF signaling is sef (similar expression to fgf genes). Sef is a transmembrane protein that interacts with FGFR1 and decreases the phosphorylation of FGFR1 and FRS2 and reduces FGF-induced cell proliferation. This leads to an inhibition of FGFR1 tyrosine phosphorylation and consequently a reduced phosphorylation state of molecules involved in the signaling of FGFR1, such as Raf-1, MEK 1/2, and ERK [75]. 

## 4. Hetero-Oligomerization with Receptor Mutants

Certain tissues express, besides the endogenous full length protein, truncated/shorter variants of the RTK that could affect its activity and the relative signaling. In particular, expression of inactive deletion mutants could determine the formation of hetero-oligomers, which could result in deficient RTK receptors. In fact, the variants would act as dominant negative mutants and inhibit the activity of the heterodimer or hetero-oligomer [1]. Some truncated forms of RTKs might be the product of genomic rearrangements in cancer; however, another possibility is the generation of truncated RTKs by alternative mRNA splicing [76]. Interestingly, a truncated form of FGFR1 was created, lacking the cytoplasmic domain containing the KD and tyrosine residues. Still this variant was able to bind different types of FGFRs (FGFR1, FGFR2 and FGFR3). Ligand binding to the FGFR was not able to trigger the activation of the signaling pathway regulated by the full length RTK. In fact, the mutated form of FGFR1 formed non-functional heterodimers because it behaved as a dominant negative mutated form of FGFR, inhibiting the biological action of FGFs [77]. Indeed, when the deletion mutant of FGFR1 was overexpressed in *Xenopus oocytes*, the mobilization of intracellular calcium was completely blocked due to the non-functional heterodimer [77]. Other studies, in order to understand more about FGFR signal transduction and the role of FGFs in development, have been conducted. In fact, Li and colleagues studied the tyrosine kinase activity of a *Xenopus* FGFR by using FGFR mutants that presented either a point mutation in the ATP-binding site or a truncation of the cytoplasmic domain. Indeed, they discovered a negative action of these receptors on the transduction of the signaling and in the processes of mesoderm formation of *Xenopus* embryos [78]. Similarly, a specifically designed FGFR1 mutant was proved to be able to inhibit the signaling of FGFR and the proliferation of vascular smooth muscle cells [79]. Interestingly, a mutated form of FGFR4, which does not contain the C-terminal domain of the RTK and is a soluble isoform, is expressed by epithelial breast cancer MCF-7 cells. This mutant is generated after the failure of splicing of intron 4, which determines the presence of an in-frame premature stop codon. However, the modality of action of this FGFR4 mutant is based on its release in the conditioned media and the modulation of the FGF action, therefore abrogating the effect of the FGF1-induced MAPK phosphorylation and prolactin gene activation [80]. Intriguingly, a connection between the downregulation induced by the expression of an RTK mutant and the degradation of the full length form has been detected. In fact, Ser252Trp FGFR2 mutation determined the constitutive downregulation of FGFR2 based on its internalization and degradation rather than on its mRNA levels [81].

## 5. RTK Cleavage

RTKs are subjected to cleavage and generation of fragments due to the action of caspases and proteases or by following alternative splicing. In particular, RTKs undergo several proteolytic cleavages operated by secretases that generate an extracellular domain, which is released in the extracellular space, and the intracellular domain that is present in the cytoplasm [76]. RTK cleavage determines the receptor downregulation as it decreases the number of full-length RTKs that can bind ligands and become activated. Recently, two interesting studies on the action of proteases on the cleavage of FGFRs have been published [82,83]. Dixit and colleagues proved that the presence of extracellular FGFR fragments can affect the function of ligand-induced FGFR signaling during cell movement. In fact, they discovered that FGFR 1, 3, and 4 were processed by ADAM17, a member of the zinc protease superfamily, as a constitutive shedding process or after phorbol ester-induced processing. In contrast, FGFR2 cleavage was mediated by ADAM10 after stimulation of calcium release by ionomycin. The soluble forms of FGFRs were able to act as antagonists and to avoid the interaction between the ligands and the full-length forms of the RTKs. Indeed, the soluble form of FGFR2 inhibited the FGF7-induced epithelial cell migration of HaCaT cells during wound healing assay. This effect was not induced when a catalytically inactive mutant of ADAM10 was expressed [83]. In support of this, extracellular soluble forms of FGFR1 in the adult retina regulated the bioavailability of FGF2, and in this case, the neurotrophic activity during neuronal degeneration was affected [84].

The mechanism explaining how the fragments of FGFRs may regulate the function of other RTKs has been discovered by Hanneken and colleagues. They studied the inhibition of cellular proliferation and in-vitro angiogenesis induced by the competition of FGFR1 soluble fragments with FGF2 ligand. Furthermore, in this case, the cleavage of FGFR1 was due to a metalloprotease, and its production was tightly regulated. Interestingly, the stimulation with FGF2 increased the shedding of FGFR1 ectodomain, and this effect was dose-dependent, whereas the usage of metalloprotease inhibitors inhibited the release of the FGFR1 ectodomain. Similarly, the effect of the treatment on the RTK cleavage was dose-dependent. Furthermore, they discovered that activators of protein kinase C, such as TPA, enhanced shedding of FGFR1, whereas this was blocked by specific inhibitors of FGFR tyrosine kinases, therefore affecting ERK signaling [82].

## 6. Clinical Aspects of RTK Downregulation

Activity of RTKs can be downregulated also through the action of antagonist ligands or molecules able to bind to the extracellular site of the receptor where the ligands normally bind, at the ligand-RTK interface or at the intracellular domain, usually at the KD [85]. An antagonist counteracts the action of a natural ligand, an agonist, partial agonist, or an inverse agonist. There can be different types of antagonists, based on their mode of action. There can be competitive antagonists (competing for the binding to the receptor or binding to an allosteric site and determining a conformational change that alters the receptor ability to bind the ligand), noncompetitive antagonists (that bind to a different site of the receptor), and irreversible agonists (where the receptors are covalently modified) [86].

Antagonist ligands are biological molecules or chemical drugs that block or dampen a biological response by binding the receptor and inhibiting the normal RTK activation and relative signal transduction. Interestingly, ligand-derived antagonists produced through protein engineering have been developed and used as potential cancer therapeutics. For instance, a developed mutant of angiopoieitin-2 ligand retained the ability to bind the receptor Tie2 but negatively influenced the ligand multimerization and RTK dimerization and activation. The angiopoietin2-derived Tie2 antagonist strongly inhibited Tie2 phosphorylation, affected endothelial capillary tube formation, and influenced cell invasion [87].

In relation to FGFR activity, several small molecule tyrosine kinase inhibitors (TKI), classified as antagonist molecules, target the ATP binding pocket of RTKs and are under preclinical and clinical development or have been accepted for cancer treatment. They can be classified as non-selective FGFR TKIs (which can act on several receptors) and selective FGFR TKIs. Among the multi-target TKI inhibitors, dovitinib, lenvatinib, lucitanib, ponatinib, nintedanib, regorafenib, and pazopanib have been reported [88,89,90,91,92,93]. Several molecules classified as selective FGFR TKIs have been discovered and are mainly directed at the RTK intracellular domain. Among these, AZD4547, BGJ398, and JNJ-42756493 are pan-FGFR inhibitors and may be used in patients who suffer from different types of cancer related to FGFR1 altered function [94]. In contrast, a molecule that binds at the extracellular domain of the RTK and allosterically inhibits its activation is SSR128129E [95,96]. Other FGFR inhibitors are TAS-120, ARQ-087, JNJ 42756493, DEBIO 1347, BAY-1163877, and FGF 401 [97,98,99].

Moreover, antibodies and antibody fragments can be FGFR blockers. The main advantage is that exclusive members or a specific isoform of a receptor can be targeted by recognition of an extracellular epitope of cell surface proteins. For instance, the molecule scFvD2-Fc may inhibit FGFR1 activation and signaling by acting either alone or in combination with the cytotoxic drug monomethyl auristatin E [100]. Interestingly, a short peptide (VYMSPF) was able to inhibit the activity of FGFR1 and to block the receptor in a dose-dependent manner. Both this peptide and the MQLPLAT, which was able to act on several FGFR forms (FGFR1-4), were identified after phage display screening [101]. Several monoclonal antibodies have been developed in order to target FGFRs and downregulate the signaling, and two different mechanisms of action have been discovered: the blocking of ligand binding, or trap-ligand, and the prevention of receptor dimerization. Among these, MFGR1877S and FP-1039 have been considered in clinical trials, as an inhibitor of receptor dimerization and as a ligand-trap, respectively [102,103].

Furthermore, a peptide named F8, CSLNHTVNC, shared a similar binding site on FGFR1 as the ligand FGF1. In fact, it interacts at the FGF1-FGFR1 interface. Noteworthy, F8 affects FGF1-FGFR1 interaction, inhibits FGFR1 downstream signaling, and FGF1-induced cell proliferation [85].

## 7. Conclusions

The modulation of RTK activity is fundamental for all the molecular processes occurring in the cell. A fine balance between activation and inhibition or downregulation of the signaling cascade of RTKs is important in order to avoid the onset of pathologies such as cancer and alterations of cell behavior during cell development, differentiation, and metabolism. While activation of RTKs has been widely studied and several molecules implicated in the signaling pathways of the individual RTKs have been identified, less is known on the attenuation and termination of the signal, especially for FGFRs. In the case of these receptors, the main mechanisms of downregulation of the signal are ubiquitination followed by degradation in the lysosomes and inhibition of RTK due to antagonist molecules [1,13,104]. Interestingly, several mutations and alterations in FGF-FGFR have been discovered to be associated with cancer, such as aberrant expression, mutations, chromosomal rearrangements, and amplifications [104]. In fact, FGF is known to be a mitogen, and the activation of FGFRs regulates cell proliferation, survival, differentiation, embryonic development, migration organogenesis, and angiogenesis [105,106,107]. The interesting aspect of the involvement of FGFRs in several types of cancer is that many molecules, such as non-selective FGFR small molecule tyrosine kinase inhibitors (TKIs), selective FGFR TKIs, antibodies against FGFs and FGFRs, and FGF-ligand trap, have been studied as possible treatments based on the previous finding on the physiological mechanisms of RTK inhibition [108]. 

Interestingly, deregulation of FGFR signaling affects tissue homeostasis and is responsible for the onset of developmental syndromes [109]. The degree of FGFR1 activity has been linked to mitogenic and metabolic functions. Therefore, full activation of FGFR1 due to the strong dimerization of the receptor is required to elicit a mitogenic response whereas a weak FGFR activation by the transient receptor dimerization is associated with a metabolic response [110]. The generation of antagonist ligands or molecules able to regulate FGFR activity may be of relevance for the proper regulation of cellular functions.

## Data Availability

Not applicable.
